# Perspectives and experiences of healthcare providers on the response to the COVID-19 pandemic in three maternal and neonatal referral hospitals in Guinea in 2020: a qualitative study

**DOI:** 10.1186/s12913-024-10670-4

**Published:** 2024-02-21

**Authors:** Nafissatou Dioubaté, Mamadou Cellou Diallo, Cécé Maomou, Harissatou Niane, Tamba Mina Millimouno, Bienvenu Salim Camara, Telly Sy, Ibrahima Sory Diallo, Aline Semaan, Thérèse Delvaux, Lenka Beňová, Abdoul Habib Béavogui, Alexandre Delamou

**Affiliations:** 1 Centre National de Formation et de Recherche en Santé Rurale de Maferinyah, Forécariah, Guinea; 2 Service de Maternité de l’Hôpital National Ignace Deen, Conakry, Guinea; 3 Service de Maternité de l’Hôpital Régional de Mamou, Mamou, Guinea; 4 Institut de Nutrition et de Santé de l’Enfant, Hôpital National Donka, Conakry, Guinea; 5 Centre National de Formation et de Recherche en Santé Rurale de Maferinyah, Forécariah, Guinea; Centre d’Excellence d’Afrique pour la Prévention et le Contrôle des Maladies Transmissibles (CEA-PCMT), Faculté des Sciences et Techniques de la Santé, Université Gamal Abdel Nasser de Conakry, Conakry, Guinea; 6 Department of Public Health, Institute of Tropical Medicine, Antwerp, Belgium

**Keywords:** Health system response, Maternal health, Neonatal health, Healthcare providers, COVID-19, Maternity ward, Neonatology ward, Sub-Saharan Africa, Guinea

## Abstract

**Background:**

The COVID-19 pandemic has adversely affected access to essential healthcare services. This study aimed to explore healthcare providers’ perceptions and experiences of the response to the COVID-19 pandemic in three referral maternal and neonatal hospitals in Guinea.

**Methods:**

We conducted a longitudinal qualitative study between June and December 2020 in two maternities and one neonatology referral ward in Conakry and Mamou. Participants were purposively recruited to capture diversity of professional cadres, seniority, and gender. Four rounds of in-depth interviews (46 in-depth interviews with 18 respondents) were conducted in each study site, using a semi-structured interview guide that was iteratively adapted. We used both deductive and inductive approaches and an iterative process for content analysis.

**Results:**

We identified four themes and related sub-themes presented according to whether they were common or specific to the study sites, namely: 1) coping strategies & care reorganization, which include reducing staffing levels, maintaining essential healthcare services, suspension of staff daily meetings, insertion of a new information system for providers, and co-management with COVID-19 treatment center for caesarean section cases among women who tested positive for COVID-19; 2) healthcare providers’ behavior adaptations during the response, including infection prevention and control measures on the wards and how COVID-19-related information influenced providers’ daily work; 3) difficulties encountered by providers, in particular unavailability of personal protective equipment (PPE), lack of financial motivation, and difficulties reducing crowding in the wards; 4) providers perceptions of healthcare service use, for instance their fear during COVID-19 response and perceived increase in severity of complications received and COVID-19 cases among providers and parents of newborns.

**Conclusion:**

This study provides insights needed to be considered to improve the preparedness and response of healthcare facilities and care providers to future health emergencies in similar contexts.

**Supplementary Information:**

The online version contains supplementary material available at 10.1186/s12913-024-10670-4.

## Background

The SARS-CoV-2 and related disease (COVID-19) is a rapidly evolving pandemic, which, along with its associated prevention and control measures, have globally led to adverse effects on maternal and newborn healthcare use, provision and quality [1, 2]. During the pandemic, the adverse effects of COVID-19 were not limited directly to maternal and newborn morbidity, mortality, or respiratory complications resulting from infection with the SARS-CoV-2 virus, but also encompass indirect effects [3–5]. For example, fear of infection while using health facilities, transportation bans and lockdowns prevented pregnant women from traveling to healthcare facilities to give birth, and were unable to seeking essential healthcare services, such as antenatal and postnatal care [6, 7]. From the healthcare provision perspective, disruptions of health service availability were document, including the closure of outpatient clinics because the services were not deemed to be essential, the closure of labor wards because of staff shortage as a result of infection or because of the reallocation of maternity staff to provide COVID-19 care [6, 7]. These factors collectively had negative consequences on the wellbeing of pregnant women and their babies and their ability to seek essential services during the pandemic [6, 7].

Health systems globally have faced a rapid increase in health care demand due to the COVID-19 pandemic. When health systems are overwhelmed, mortality from infections and vaccine-preventable conditions increases [8]. Public confidence in the ability of the health system to meet basic needs in health facilities is essential and enables continuity of public adherence to seeking emergency obstetric care and neonatal intensive care [9, 10]. The success of the response to COVID-19 on limiting the disruption in maternal and newborn care services differed substantially across countries [11]. In addition, the impact of COVID-19 on the availability and quality of maternal and newborn health services depends on the scale of the epidemic (number of reported cases and deaths) in a given area, as well as the preparedness of the health system (organization, availability of skilled health personnel, sufficiency of personal protective equipment [PPE]).

Tertiary referral hospitals for maternal and newborn care play a crucial role in health systems as they provide care to some of the most complicated obstetric and neonatal cases, including by receiving referrals [6, 12–14]. Due to the high volume of patients they serve, the role they play in a health system and their capacity of expert staff, these hospitals seemed to be the first to develop protocols, train staff, encounter cases of COVID-19 among pregnant women, and trial processes on how to provide good quality maternal and neonatology care during the pandemic [12, 14–16].

Guinea is a country that carries a high burden of maternal mortality, with a ratio estimated at 553 deaths per 100,000 livebirths in 2020, and neonatal mortality at 31 per 1000 livebirths [17]. The progression of COVID-19 in Guinea has been characterized by a gradual increase in cases between March and December 2020, with a total of 14,840 confirmed cases including 54 deaths during this period [18–20]. However, among the decisions made and the strategies implemented during the response to the COVID-19 pandemic, some important aspects were not considered. These include healthcare providers’ needs to timely access to accurate information, and involving healthcare providers in decision making and management of a health crisis of this scale [21, 22]. Study in Brazil showed that infection with COVID-19 during pregnancy increases the risk of mortality [23]. However, such data does not exist from Guinea. A quantitative study conducted in three hospitals in Guinea looking at aggregate routine data showed an increasing trend in maternal and neonatal deaths during the COVID-19 pandemic [24]. These shortfalls occurred despite the country’s previous experience with the Ebola Virus Disease (EVD) epidemic, which presented learning opportunities to overcome difficulties related to affected countries’, including Guinea’s, preparedness and response to epidemics [18, 25].

This study was part of a multicenter mixed-methods research project conducted in four sub-Saharan African countries: Guinea, Uganda, Tanzania and Nigeria [26]. The multicenter study aimed to understand the perceptions, views, and experiences of healthcare providers in providing care to women and newborns in large referral hospitals during the first year of the COVID-19 pandemic. The objective of this paper was to explore healthcare providers’ perceptions and experiences of the response to the COVID-19 pandemic in three referral maternal and neonatal hospitals in Guinea.

## Methods

### Study design and duration

We conducted a longitudinal qualitative study between June 1 to December 31, 2020. The prospective nature of the study consisted of interviewing several respondents in each included hospital repeatedly over four rounds of data collection conducted three to 4 weeks apart.

### Study setting

This study was conducted in three referral hospitals: two maternity wards (Hôpital National Ignace Deen (HNID) in Conakry and Hôpital Régional de Mamou (HRM) in Mamou) and the neonatology ward of the Institut National de Nutrition et de Santé de l’Enfant (INSE) in Conakry. The HNID maternity ward, located on the Kaloum peninsula in Conakry, is among the apex referral services in the country’s health system pyramid, with about 6000 births per year. The maternity ward of the HRM, located 275 km northeast of Conakry, is at the top of the health pyramid in the Mamou region, with an average of 3600 births per year. INSE is located in Kaloum, Conakry within the Donka National Hospital compound and is the sole intensive neonatal care referral ward in the country, with an average of 2100 neonatal admissions per year [20]. At the time of this study, the Donka National Hospital maternity ward was closed for renovation, meaning that all neonatal admissions at INSE were referrals from other facilities or self-referrals.

### Sampling and recruitment

Participants (i.e. healthcare providers) were purposively recruited to capture diversity of professional cadres (medical doctor, nurse, midwife), seniority levels (professor/head of ward, junior providers, interns) and genders, to capture a range of perspectives and experiences, allowing for maximum variation in the information collected, and until information saturation was achieved [27]. Saturation was defined to be reached after the lack of generation of new information in the interviews, among different types of respondents and different rounds, and considering the stabilization of the COVID-19 situation in the country. We conducted IDIs in the three study sites with two types of respondents: first, regular respondents i.e. managers and directors of the wards and their assistants, as well as the supervisors of healthcare service activities in the two maternity wards and INSE, who were interviewed regularly during each of the four rounds; second, with irregular respondents who included healthcare providers, namely medical specialists and trainees, midwives and state registered nurses and who were interviewed only once during the study.

### Data collection

A total of *n* = 46 in-depth interviews were conducted during the study period. Four rounds of interviews (around 12 IDIs planned per round over the three study sites) were conducted over 6 months namely June, July, August–September, and November–December 2020. We developed and used an interview guide with 18 main questions, to capture changes and adaptions to healthcare facility infrastructure, hygiene, care provision, data capture and community care-seeking behaviors in light of the COVID-19 pandemic. We also explored respondents’ personal experiences with stress and access to personal protective equipment. The interview guide was adapted to each facility context, respondent, and round of data collection. The interviews were conducted by an experienced qualitative researcher (ND) in French and audio-recorded. The necessary infection prevention and control measures were respected to protect the respondents and researchers from risk of infection transmission. In HRM, the first seven interviews were carried-out in person and the rest on the phone.

### Data analysis

The audio-recordings were transcribed into French. From these, some quotes were translated into English, while pseudo-anonymizing. Qualitative data analysis was performed by developing an initial coding scheme, guided by the study objectives, after carefully reading and re-reading the transcripts. The coding tree was developed based on the main multi-country study, with specificities elaborated in relation to concepts specific to Guinea (e.g. links to Ebola outbreak). The coding tree included four mother-codes: changes resulting from COVID-19, adapting to changes, provider behavior towards patients, and management of COVID-19 cases. Coding was done by ND with N-Vivo v12. We then used an inductive approach of content analysis by identifying new themes in the data. An iterative process was used involving researchers and social scientists from different backgrounds (ND, BSC, AS, TD) to enhance the validity of the findings. The data were summarized by theme and sub-theme per study site and chronologically by data collection rounds, presented in a table, and narratively summarized.

## Results

A total of 46 IDIs were conducted (11–12 IDIs per round) with 18 respondents (10 men and eight women), considering the professional categories existing in the three sites (Table 1). Interview duration ranged between 18:37 and 60 minutes, depending on the round of data collection.

**Table 1:** Description of interviews and study respondents in the four rounds in the three study sites (*n* = 46 interviews with 18 respondents)

Study site	Respondent types	In-depth individual interviews (IDIs) during the four rounds				Total IDIs per facility
	*Round*	*1st*	*2nd*	*3th*	*4th*	
	*Dates (DD/MM/YY)*	*08/06/20* *24/06/20*	*04/07/20* *27/07/20*	*03/08/20* *03/09/20*	*02/11/20* *30/12/20*	
Maternity ward of HNID	Regular respondents *(*OB/GYN *and midwife)*	3	3	3	2	15
	Irregular respondents (OB/GYN, Year 4 trainees, midwife)	1	1	1	1	
Maternity ward of HRM	Regular respondents (Medical Doctors)	3	2	3	3	15
	Irregular respondents (midwives)	1	1	1	1	
Neonatology ward of INSE	Regular respondents (pediatricians and nurse)	3	3	3	3	16
	Irregular respondents (pediatrician, Medical Doctor, nurse)	1	1	1	1	
**Total**		**12**	**11**	**12**	**11**	**46**

*HNID* Hôpital National Ignace Deen, *HRM* Hôpital Régional de Mamou, *INSE* Institut de Nutrition et de Santé de l’Enfant

We found that respondents had different perceptions related to their facilities’ response to the COVID-19 pandemic (Table 2). We identified four main themes: 1) Coping strategies and reorganization of care provided in the wards as a result of COVID-19, 2) healthcare providers’ behavior adaptations, 3) Difficulties encountered during the pandemic and 4) the perception of providers on the use of healthcare services.

**Table 2:**
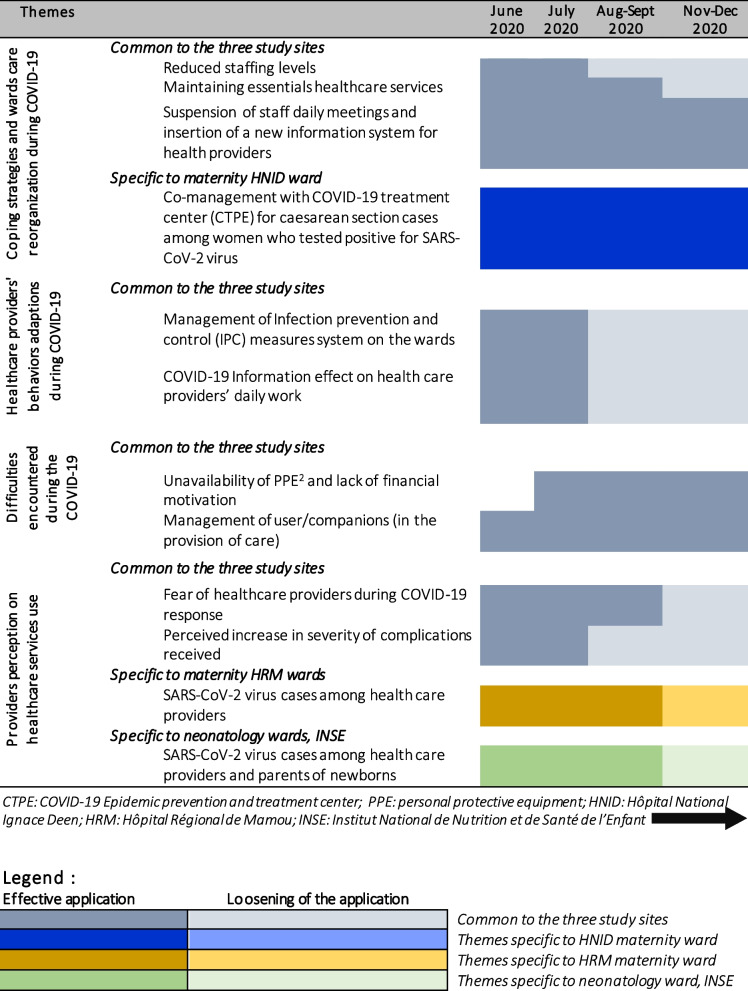
Evolution of healthcare providers’ perceptions of and experiences during the response to COVID-19 over the four rounds of IDIs, by study site, Guinea, 2020

### Theme 1: coping strategies & care reorganization during the COVID-19 pandemic

In the three study sites, respondents reported both common and site-specific perceptions regarding this theme and the related sub-themes (Table 2).

### Strategies and care reorganization common to the three study sites

#### Reduced staffing levels

According to respondents, the first strategy applied in the wards in response to the COVID-19 pandemic was to reduce the number of healthcare providers. This reduction consisted of the discharge of trainees and volunteer staff, leading to an internal reorganization of services. The reorganization resulted in a rotation system for regular providers from eight-hour to a 24-hour shift (8 to 24 h). This strategy aimed to promote physical distancing to reduce the risk of exposure and contamination of the SARS-CoV-2 virus among healthcare providers and patients. They noted also that the reduction in staffing numbers during the early phase of the pandemic had not led to difficulties because during this period (June to July) there was a perceived decrease in the number of patients in the wards.

*“We received a circular from the Ministry of Health asking to release all the trainee staff, it was to avoid overloading the facility..., we also released early the trainees coming from schools of health and universities.”* (Doctor N°1, Neonatology-INSE, Round 1)

However, after the second round of interviews, healthcare providers felt overwhelmed by workload due to the progressive resumption of patient attendance. Thus, at their request, there was a gradual reintegration of the trainees and volunteers to facilitate regular providers’ daily works in the wards.

#### Maintaining essential healthcare services

According to the participants, the activities of their wards were essentially centered on the management of obstetric and neonatal emergency cases between June and July 2020.This was accompanied by a set-up of an admission triage system that allowed for the identification of urgent cases and their immediate management. The other cases (routine consultations or uncomplicated deliveries) were either postponed or redirected to nearby public health facilities (first- and second-line health facilities). The reasons reported were to reduce the workload of senior providers by decreasing the number of patients and to reduce the risk of exposure to SARS-CoV-2 for both healthcare providers and patients (decrease in waiting time and length of care). In addition, the providers stated that they had the impression that the quality of care services (especially reception) had improved and they attached this to their long experience of medical practice.

*“As far as the quality of the work in the delivery room or any other unit is concerned, the quality has not decreased because there are attendants who have an average of 15 to 20 years of experience. Although the number of staff has been reduced, everyone (still working) knows what to do. In fact, there were even women who have received care during COVID-19 who appreciated the quality of the work, especially in the delivery room.”* (Doctor N°2, HRM, Round 1).

According to the participants, the activities in the wards mainly focused on the management of obstetric and neonatal emergencies between June and July 2020.

Around August, at the Conakry sites, the suspended activities in the hospitals (routine consultation and management of non-urgent cases) were gradually resumed due to increasing patient volume. Then, with some restrictive measures lifting nationally, HRM joined the two others sites to gradually resume activities. According to the respondents, the situation returned to normal (before COVID-19 period).

#### Suspension of staff daily meetings and introduction of a new information system for health providers

Respondents noted that to reduce the risk of SARS-CoV-2 exposure among healthcare providers, the frequency and number of participants in regular in-person staff meetings was gradually decreased until the meetings were suspended altogether between March and June 2020. In Conakry (HNID maternity ward and INSE neonatology ward), new communication channels were explored through virtual meeting platforms (such as WhatsApp and Zoom) to counter the lack of opportunities to meet within the teams. Used internally (within the wards), these platforms have not only allowed for better information sharing but also to allow all staff to be at the same level of information on the functioning and management of the pandemic in the services including the evolution of the pandemic in the country. Respondents also emphasized that they appreciated these virtual communications because they allowed them to easily participate in meetings, saved time, allowed them to seek other opinions on cases received (at any time) and to quickly establish adequate management because decisions were not delayed. They also mentioned that they wanted to keep this new communication method, which is why its utilization continued throughout the entire study period.

"What we need to remember from this period [referring to the COVID-19 pandemic] for example this platform (referring to WhatsApp group) that is there, it is an innovation, ..." (Doctor N°3, HNID, Round 4)

Taking into account the conditions required to use these platforms (having a computer or Android phone, and access to internet connection), in the HRM site, an alternative was considered which consisted of creating of a local response committee (at the hospital level) for the COVID-19 pandemic. This committee, which was composed of a limited number of participants, met weekly to provide updates on the pandemic (sharing experiences, adapting the case management protocol, or establishing new guidelines in the region) and disseminate this information to each hospital ward through the manager (heads of ward) and supervisors to the healthcare providers.

### Strategies and care reorganization specific to HNID maternity ward

#### Co-management with COVID-19 treatment center (CTPE) for caesarean section cases among women who tested positive for SARS-CoV-2 virus

During the response, the epidemic prevention and treatment center of Donka hospital (CTPE) had an agreement with HNID maternity ward to provide care to pregnant women who were infected by SAR-CoV-2 virus and had an indication for cesarean section. Transferred by ambulance with a CPTE staff, these women were immediately taken to the operating room specially dedicated for these cases by the HNID staff. Then, after the cesarean section, mothers and their newborns returned to the CTPE for continued post-operative care. Thus, this co-management continued until the last round of interviews in December 2020.

For other infected pregnant women, a providers’ team (composed of an obstetrician-gynecologist, a midwife and a nurse) was available in the CTPE to provide care services.

*“Being the only [functional] reference service at present, we are at the disposal of the ANSS [Agence National de Sécurité Sanitaire] and the infectious diseases services to support them whenever necessary [to perform the caesarean section on pregnant women who have tested positive for the COVID-19 virus] ... But these women do not stay with us here, yes, yes! After the surgery they and their babies return to the CTPE, .... CTPE of Donka ». (*Doctor N°1, HNID, Round 1)

## Theme 2: healthcare providers’ behaviors adaptations during COVID-19 response

In this section, we focus on providers’ behaviors toward these two sub-themes: management of Infection prevention and control (IPC) measures system and how COVID-19-related information influenced healthcare providers’ daily work.

### Healthcare providers’ behaviors common to the three study sites

#### Management of infection prevention and control measures system on the wards

When the first cases of COVID-19 were noted in the country (March 2020), IPC kits were made available in every health facility to reduce the risk of infection. These included hand washing kits, personal protective equipment (PPE) including masks, caps and disposable aprons for deliveries, and thermometers.

In the study wards, although being in PPE was unusual and sometimes uncomfortable, respondents recognized that adherence to and compliance with IPC measures was their primary protective measure in the response to the COVID-19 pandemic. These measures included proper use of masks (nose and mouth fully covered), hand washing (including use of hydro-alcoholic gels) before and after each medical procedure, full use of PPE in delivery and hospital rooms, limiting the number of people (including providers and users) in a room, and physical distancing. To further reduce risk, some beds were removed from hospitalization rooms and also seats from the waiting rooms. Strict adherence to these measures was mentioned during the first three rounds of interviews. However, during our last visit, a slackening was reported following the decrease in COVID-19 diagnosed cases and the lifting of restrictive measures throughout the country.

In addition, security guards whose primary focus before the pandemic was to ensure security, were requested to enforce the application of infection prevention and control measures by healthcare providers, patients and companions. Security guards were located in front of the hospital entrances, and the wards as well. Thus, each person who wanted to enter the wards was subjected to a double control (hand washing, verification of the correct wearing of masks, temperature taking). Security guards were also responsible for ensuring compliance with the measure consisting of limiting the number of companions to one companion per patient in the wards.

#### COVID-19 information effect on health care providers’ daily work

For some participants, the information they received regarding COVID-19 management enabled them to adopt attitudes aiming at controlling the risks of exposure and contamination and to feel more confident and better equipped to deal with the pandemic in their respective wards. These information included COVID-19 case definitions (warning symptoms), infection prevention and control measures, daily reports on COVID-19 situation at the national level, management protocol of suspected cases in delivery and hospitalization rooms.

For others, although they had received information on COVID-19 management, they would have liked to have received training or refresher courses adapted to this health crisis. Because, according to them, health crises are different from each other, referring to the management of the Ebola virus disease (EVD) epidemic that the country had experienced earlier (2014–2016).

But, between August and December 2020, there was a gradual decrease in information sharing frequency, which according to them, allowed them to infer the end of the pandemic in the country. This inference led to a slackening of compliance with IPC measures among healthcare providers.

## Theme 3: difficulties encountered by providers’ during COVID-19

The difficulties encountered by healthcare providers were summarized as unavailability of PPE, lack of financial motivation, and difficulties in managing users and companions.

### *Difficulties of the COVID-19* during the COVID-19 *common to the three study sites*

#### Unavailability of PPE

***O***pinions were divided on the availability of PPE. For some respondents (health providers), the first months of the pandemic (until June 2020) were characterized by availability of PPE in sufficient quantities. Healthcare providers reported not having to worry about PPE shortage when they were in the hospital because their hospitals supplied them “*in sufficient quantity and quality”* daily and free of charge. But this view was not shared by the wards’ supervisors, who noted difficulties (from the beginning of the response) in supplying providers with PPE during working hours. Starting July onwards, they reported (supervisors and providers) a decrease in this supply capacity of PPE in their wards, leading some providers to buy their own disposable or re-usable facemasks (e.g.: local tissue facemasks).

*« the ministry never supplied us with PPE, the hospital sometimes supplies us (the maternity ward) with masks, not more … When you give 150 masks, it’s not for half a day, we do not wear the masks for 2 or 3 days only … we wear them until they turn black”. (*Doctor N°1, HNID, Round 4).

#### Lack of financial motivation

Respondents also noted the lack of motivation related to financial compensation (in form of a bonus) in view of the increased risks for themselves and their family members. According to them, this point should also be important since they are always the first to be exposed to infectious diseases and they put their relatives (families and proximities) at risk. They also mentioned that some of their colleagues refused to come to the wards because the COVID-19 exposure risk was too high for them. These views were widely shared among the nursing and midwifery team for most of the study period (July to December 2020).

*“There were some people [referring to these provider colleagues] who refused to come to the ward, but we came, until now some don't come. We who agreed to come, we didn't get a pay raise, we didn't get a bonus [with a shrug]”.* (Nurse N°2, Neonatology-INSE, Round 2).

#### Difficulties reducing crowding in the wards

In order to reduce the risk of transmission of the virus, the strategy of limiting the number of companions per patient was introduced. Indeed, visits to hospitalized patients were forbidden and only one or two companions per patient were allowed in the premises of the ward, particularly in outpatient clinics, post-delivery rooms and especially in patients’ hospitalization room (for mothers and newborns). However, the staff was always sending away visitors and companions whose presence was not necessary, despite their efforts through many (daily) sensitizations on the risks of transmission of COVID-19 to the latter. This created a source of palpable tension between staff members (specially supervisors and security guards) and patients, companions or visitors. This tension sometimes made it difficult to collaborate (exchange information) in patients’ management and follow-up. According to the respondents, the main reason for this situation was related to the socio-cultural context of the country, because both events (illness or birth) led to large numbers of people who close to patients (parents or friends) visiting the hospital, regardless of the location.

*« This is one of the big problems of the ward. People have the culture of coming in large numbers with the patients, despite the COVID-19. We don't need three people to accompany one patient”.* (Doctor N°3, HNID, Round 4).

## Theme 4: providers’ perceptions on healthcare services use

This theme describes healthcare providers’ fear during the response to COVID-19 and how they perceived the pandemic’s impact on services use.

### Perceptions of healthcare service use common to the three study sites

#### Fear of healthcare providers during COVID-19 response

According to respondents, in addition to the general fear of the COVID-19 pandemic, healthcare providers faced another concern connected to the increase in cases referred to the wards. This concern was heightened by the fact that some of these patients came from private health facilities where COVID-19 positive cases were reported. In addition to the virus’ mode of transmission, the proximity of the CTPEs to the study sites, the difficulties of managing users and companions, shortage of PPE, lack of COVID-19 tests accessibility to providers and patients onsite, and the long waiting time to obtain the results (at least 24 hours) worsened this fear, which in turn generated a palpable state of stress among healthcare providers. This was reported during the first three rounds of interviews and started to decrease in November due to the lifting of restrictions and the healthcare utilization resumption of wards and hospitals.

Also, from the beginning of the pandemic, some respondents mentioned that they received many phone calls from patients for both minor and major health problems without this health crisis situation (COVID-19 pandemic), patients would have come to see them at the hospital premises. For some providers, they encouraged these calls for several reasons, notably to continue to be close to the patients because the proximity of some COVID-19 treatment centers to the wards (INSE and HRM) and the previous negative experience of patients with large hospitals during the EVD epidemic demotivated them to come to the premises. They also saw the calls as a way to make their daily work easier (deducting patients with minor health problems) and finally as a more economical way for patients’ to get quick answers to their health problems.

*"A mother who has to spend 20,000 GNF [US$2.29] to bring her child here, if she uses 5,000 GNF [US$0.57] of phone credit to call the doctor and get advice from him, I think that's a gain for the mother. If it turns out that this child is not sick, we can treat him on an outpatient basis. Sometimes it's the transportation that tires them [the patient and the family],* .... "(Doctor N°1, Neonatology- INSE, Round 4).

But for others respondents, it was seen as an opportunity to limit patient contact and thus reduce the infection risk. Although different reasons were given, the underlying reason was their negative experience during the response to the EVD epidemic because some (licensed) providers did not come to work during the COVID-19 pandemic response period.

*“since the time of EBOLA we are here, we come to the service, we have to take measures to avoid the infection… There were people who refused to come to the service, …, until now some don't come. We who accept to come, we are all afraid of being contaminated, but we take all the precautions not to be contaminated, it is not easy for us*". (Nurse N°2, Neonatology- INSE, Round 2).

#### Perceived increase in severity of complications received

Some respondents reported facing a high number of complications such as respiratory distress for newborns, eclampsia or uterine ruptures for women, all due to delayed care. Reasons cited for these delays included travel restrictions, reliance on traditional medicine or self-medication, and their negative experiences using public services during the EVD epidemic. According to participants, travel restrictions during the COVID-19 pandemic made it difficult for patients to be transported because most of them used common means of transportation, which were rarely available at that period. Therefore, to avoid any hassle, the first recourse of patients was traditional medicine (plant, leaf or bark), then self-medication. If not improved, they used public health services or private structures of proximity which administered oxytocin more than necessary. Finally, in order to get to the referral services, the many checks carried out by the authorities during the journey did not facilitate access to these referral services for the users. The participants emphasized that the negative experience of the EVD epidemic influenced this late choice to use the services (especially public health facilities). Thus, in the end, these referral services received these patients with more or less severe complications, sometimes even involving the patient’s vital prognosis. These two situations were mainly observed between the first two interviews.

*“It is the users who no longer come, they have abandoned the referral hospital in favor of the [private] clinics. So, they use oxytocin, that the woman takes but she cannot give birth. She stays a long time and finally she comes to the [referral] hospital and this creates problems”.* (Midwife N°2, HRM, Round 3).

### Use of healthcare services specific to HRM maternity ward & INSE neonatology ward

#### COVID-19 cases among healthcare providers and parents of newborns

During the COVID-19 pandemic, COVID-19 positive cases were recorded among HRM and INSE providers. These positive cases occurred as a result of contact with patients and companions who came for care on the premises of the services. Providers who tested positive were subsequently hospitalized in their respective CTPEs. According to the respondents, this situation created mistrust and tension between users and providers. It also made collaboration for care between the two groups difficult because it was perceived as a stress source. The quote below supports this statement:

*« In all, I believe there have been 15 cases [referring COVID-19 cases among healthcare providers the HRM] ..., but in my maternity ward, we have had two cases that were managed at the CTPE [of Mamou]. So, this had an impact on the functioning of the service, as it created a certain psychosis.”* (Doctor n°1, HRM, Round 3).

At INSE, in addition to SARS-CoV-2 infection among health staff, other cases were recorded among parents of hospitalized newborns, which increased tension among the team of providers and also among other parents. This contributed to reduced care utilization, as some parents requested early discharge. This situation led to the opening of specific rooms for newborns whose parents were positive for COVID-19. These rooms were also used to receive the newborns of mothers tested positive who had given birth (by normal delivery or caesarean section) in the CTPE premises. After delivery, these newborns were automatically referred to the INSE neonatology unit for care and follow-up during all mothers’ stay at the CTPE.

#### Reflexivity

The lead researcher (ND) is a female medical doctor holding a Master of Public Health and with five-years working experience as a researcher, with proven experience in collecting and analyzing qualitative data part of ethnographic studies in Guinea. Her previous research involved a study on nosocomial infection with Ebola Virus Disease, where she was present in the hospital for full weeks to observe patients and care providers. This predisposed her to assume that respondents in this study would prefer to focus on themes related to fear of infection spread, including potential tensions between provider-provider or patient-provider. However, as the study and data collection evolved, the researcher came to realize that providers were eager to discuss and delve more in-depth into other topics in the interview guide, particularly that they had reported having more information about IPC which reassured them (in contrast to Ebola), and better self-control towards patients and companions. Additionally, despite the IPC measures put in place in the hospitals and particularly in the frame of this research project, the researcher was cautious and suffered from a fear of infection with COVID-19 herself.

This study was set-up as a scientific collaboration with managers at the maternity departments of the included facilities, and the managers are included in the authorship of this paper. The heads of department (collaborators on this paper) were responsible of introducing the study to potential participants ahead of time. This collaboration was thus reflected by the engagement and commitment of facility staff to participate in the interviews, and none of the respondents who were asked to participate in the interview refused. Before the interview the researcher introduced herself to the participants and introduced the study and the objectives as part of the consent process, and room was open for questions from respondents before the start of the interview. The interviews took place in the respondents’ workplace, in a separate room with closed doors to ensure that respondents can privately and freely share their experiences and perceptions.

The tools used to collect the data were co-developed by the research team. The interview guide was not pretested, because 1) of the urgent need to begin data collection as soon as possible and capture the situation prospectively, and 2) this is a qualitative dynamic study which took place in two different contexts, and we amended the guide as the study evolved. Data collection was accompanied by field notes taking during and after the interviews by the lead researcher. The notes were being discussed during bi-weekly meetings with the research team as a starting-point for the analysis, and were also used to edit the interview guide for the future rounds of interviews. These meetings were also attended by the heads of departments who commented on the validity/relevance of the content of the interviews. Transcripts were not shared with participants for comments or corrections. Instead of this time-consuming task, regular respondents were given oral feedback and summaries of their previous interviews during the subsequent data collection rounds, which allowed them to validate the preliminary data and provide feedback/corrections as needed.

## Discussion

This study provides useful information on the adaptive response of maternal and newborn referral services during the COVID-19 pandemic in Guinea. Thus, many strategies were explored in the three wards, that including adapting and reorganizing in care provision, interactions (between providers and/or between providers and patients) and difficulties encountered. It describes also healthcare providers’ perceptions (quality of care provided, fear etc.…) and their experiences in the response to infectious diseases (e.g., EVD). This reporting followed how these evolved over the time (first year of COVID-19 pandemic), context (Conakry and Mamou) and specificity of each of these large referral hospitals during COVID-19 pandemic.

Among the strategies implemented, respondents identified the reduction of health care staff as one of the most effective strategies for reducing the risk of COVID-19 spread through referral hospitals. This reduction consisted of keeping only tenured providers (removing interns and volunteers) on the wards and was in line with physical distancing efforts. A global survey conducted by Semaan et al. and a rapid review of evidence from past epidemics carried-out by Desborough et al. [1, 28] had similar findings. Despite the advantages of this strategy in preventing the disease spread and maintaining care quality improvement using only more experienced providers, it is known to be a source of mental and psychological discomfort for the retained healthcare providers (due to work overload) and for those on standby (due to their difficulties in dealing with daily life, particularly in such a pandemic context) [29, 30]. In addition, the number of patient companions was also limited (one companion per newborn and up to two per woman) and visits were prohibited during the hospital stay. This decision was made in relation to healthcare providers’ experiences in the fight against previous infectious disease outbreaks such as the EVD. Although this decision sometimes created tension between providers and users, it was beneficial not only to reduce the risk of virus spread between providers and users, but also to facilitate the daily work of regular providers. Then, some activities, such as daily staff meetings (initially used as a means of communication) and which required the gathering of more than five providers, were suspended in these hospitals. Thus, to address the lack of communication within the teams, the use of platforms such as WhatsApp or Zoom were introduced as new means of communication. This adaptation has been useful for sharing information on ward functioning and on COVID-19 pandemic evolution (especially in the hospitals and wards). The continuity of this practice will depend on its understanding and acceptance by other professional categories (midwives and nurses). Another issue is that it could mean to additional costs for providers which were not covered by the facility [31].

In the three referral hospitals, the nature and frequency of information received by healthcare providers led them to adapt accordingly. When providers were given frequent updating information, they maintained a rigorous IPC measures application as they were aware that complying with IPC measures was their main weapon against the pandemic. In contrast, when the frequency of updates decreased, providers inferred that the infection risk was decreasing or even that the pandemic was over. The lifting or loosening of certain restrictive measures (travel bans or gatherings) strengthened this perception, which favored new infected cases among healthcare providers and users, thus increasing the level of stress and distrust between the two groups. These findings highlight the need for actionable recommendations to be shared together with information about an epidemic trajectory. In addition, there is a need for staff training or capacity building as a stronger information path in function of each health crisis given the specificities of infectious disease epidemics. Ensuring the appropriate information flow, followed by actionable recommendations within an epidemic context enables for coping with uncertainties, fear, questions raising, unnecessary stress and stimulating motivations among healthcare providers [32].

The main benefit of the IPC measures put in place during the COVID-19 response was the safe environment they provided for providers to continue to offer essential care to patients. Despite this enabling environment reinforced by ward reorganization strategies, there was a decrease in the utilization of care services. In our context, this could be explained by the negative perception that users of public health services have due to their negative experience with the advent of the EVD epidemic that the country experienced in 2014–2016 [33]. The proximity of the CTPE has helped to maintain also this negative perception.

Although the solution was self-evident, telephone calls were used (via users) as a way to facilitate continuity of care. For providers, it was seen as an opportunity to limit contact, reduce the risk of virus transmission, and ensure timeliness in the healthcare delivery. However, there are many challenges associated with the use of this technology for access to healthcare, especially in health crises period such as COVID-19, as the implications of this technology are not always understood in our context. These implications can range from the stability of these networks, to the ability to pay for the prerequisites for its use (by users) on the one hand, or its standardization and understanding (by providers) to provide quality health care on the other. Galle et al. [34] also note that the use of this technology could be inequitable in many ways, excluding the most vulnerable/poor women.

The Lack of PPE was one of the difficulties encountered by healthcare providers in the response to the COVID-19 pandemic in the three hospitals. This situation was reported throughout the study period and contributed to the decreased motivation of many of them because they felt they were not adequately equipped to deal with a health crisis of this magnitude. Given the risk of exposure of this group especially in the response to infectious diseases, this availability is all the more important because it indirectly influences the quality of care (maintaining their level of motivation), it provides a safe working environment (reducing stress levels), and does not affect their economy (they will not have to pay out of pocket for resupply) [35].

### Lessons learned

The findings of this study point out several lessons that could guide the healthcare provision during the COVID-19 pandemic or any other similar health crisis. First, it could be helpful to use mobile applications (WhatsApp, Zoom, etc.) in referral facilities to support and improve communication among healthcare providers (especially nurses and midwives) by addressing potential communication gaps/delays in health emergencies and beyond while considering the financial implications for providers. However, we acknowledge that using such tools includes potential security risks and privacy concerns, especially if sensitive patient information is being discussed. Second, despite their experience with epidemics (e.g. EVD), which is relatively limited for most healthcare providers, they should have training/capacity-building courses on infection management, prevention, and control, tailored to each health emergency when it occurs. Third, there is a need to concretely employ the knowledge produced from responding to previous infectious disease outbreaks and health system shocks to elaborate comprehensive emergency preparedness and response strategies, and the health facility, district, regional and national levels. Fourth, health authorities should ensure that sufficient quantities of PPE (preventive stocks) are available in referral public facilities to maintain the level of motivation and these implications, also reduce the level of stress (avoid possibility of possible infection risk) of healthcare providers and thus avoid the resulting mistrust. Fifth, strategies to strengthen public confidence in the public health system and to control practices in private facilities should be implemented. Finally, the provision of quality healthcare services and continuity for pregnant women and their newborns should be prioritized in the response to future health crisis.

### Strengths and limitations

Despite these limitations related to the approach used (qualitative), our study allowed us to explore changes in-depth while its prospective nature was useful to observe and document changes over time. Also, the mix of perceptions of different categories of healthcare providers and the context of the study sites (two regions with these different realities) provided a wealth of data used to generate relevant evidence. However, we acknowledge that our study bears limitations including the fact that the qualitative nature of our study cannot allow the results to be generalized. Thus, the different coping strategies and reorganization of services, the effects of these changes on healthcare providers and the delivery of maternal and newborn care services identified in this study are specific to the study contexts. Future studies, particularly ethnographic studies, are needed to deepen our findings and include other regions of the country. Additionally, we did not collect information about respondent’s age, years of experience or other characteristics which could be relevant to the interpretation of the results. However, we collected and mention information on respondents’ cadres and seniority levels, which could be a proxy of age and years of experience, and were taken into consideration during the design and analysis.

## Conclusion

Our study provided insights into healthcare providers’ perceptions and experiences of COVID-19 response in referral maternal and neonatal health facilities. Despite the strategies and reorganizations that have been observed in these health facilities, this study shows that another aspect needs to be taken into account, namely the perceptions and opinions of healthcare providers who are on the front line in the process of responding to infectious diseases (pandemics or pandemics). These insights need to be considered to improving the preparedness and response of health services and care providers to future similar health emergencies.

### Supplementary Information

**Additional file 1.**

**Additional file 2.**

**Additional file 3.**

## Data Availability

All data generated or analysed during this study are included within this published article.

## Abbreviations

COVID-19: Coronavirus disease of 2019
PPE: personal protective equipment
CTPE: Epidemic prevention and treatment center
IPC: infection prevention and control
HNID: Hôpital National Ignace Deen
HRM: Hôpital Régional de Mamou
EVD: Ebola Virus Disease
NSE: Institut National de Nutrition et de Santé de l’Enfant
DIs: In-depth interviews
CNERS: Comité National d’Ethique pour la Recherche en Santé
