# Supplementary material for: Perspectives and experiences of healthcare providers on the response to the COVID-19 pandemic in three maternal and neonatal referral hospitals in Guinea in 2020: a qualitative study
Source: BMC Health Serv Res. 2024 Feb 21;24:226. doi: 10.1186/s12913-024-10670-4 (PMC10882787; doi:10.1186/s12913-024-10670-4)
Supplement: Supplementary file 1 — Additional file 1. [file 12913_2024_10670_MOESM1_ESM.pdf]

MINISTERE DE LA SANTE DE LA GUINEE

CENTRE NATIONAL DE FORMATION ET DE RECHERCHE EN SANTE RURALE DE MAFERINYAH

Préparation et riposte au COVID-19 : une enquête sur les prestataires de soins de santé dans 3 sites en Guinée (Maternités et unités néonatales)

Protocole de Recherche

Draft, Version 0.2 du 13 octobre 2020

Investigateurs

Maternité de l'Hôpital National Ignace Deen (Conakry)	**Prof. Telly SY**
Institut de Santé et de Nutrition, INSE, (Conakry)	**Dr Ibrahima Sorry DIALLO**
Maternité de l'Hôpital Régional de Mamou	**Dr Lamine DIALLO**
Co-Investigateurs	**Dr Abdourahmane DIALLO**<br>**Dr Mahamoud Sama CHERIF**
Coordinateur	**Prof. Alexandre DELAMOU**

1.

Annexe 1. Fomulaire et œrtificate de consentement libre et éclairé FORMULAIRE DE CONSENTEMENT LIBRE ET ECLAIRE

Le formulaire de consentement éclairé et le certificat de consentement devront être utilisés pour la participation à l'étude.

Titre de l'étude : Préparation et riposte au COVID-19 : une enquête sur les prestataires de soins de santé dans 3 sites en Guinée (Maternités et unités néonatales).

- **Investigateurs de l'IMT** : Lenka BENOVA, Dr Thérèse DELVAUX, Aline SEMAAN.
- **Investigateurs de Guinée:** Prof. Telly SY, Dr Ibrahima Sorry DIALLO, Dr Lamine DIALLO, Dr Abdourahmane DIALLO, Dr Mahamoud Sama CHERIF
- **Coordinateur du projet d'étude:** Prof. Alexandre DELAMOU

Introduction : bonjour, je m'appelle ***Dr DIOUBATE Nafissatou***, nous menons cette étude en collaboration avec le Centre national de formation et de recherche en santé rurale de Mafèrinyah et l'Institut de médecine tropicale d'Anvers (IMT) pour documenter l'état de préparation et la réponse à COVID-19 dans les grandes maternités et Unités néonatales de la Guinée mais aussi de trois autres pays d'Afrique subsaharienne.

Informations sur l'étude :

Nous menons cette étude pour documenter l'état de préparation et la riposte au COVID-19 dans trois sites (2 maternités et une unité néonatale) en Guinée. En plus d'autres méthodes de collecte de donnée utilisées dans cette étude, nous réaliserons des entretiens individuels approfondis. Nous estimons la durée de cet entretien à environ 45 à 60 minutes. Les résultats vont aider les autorités à mieux soutenir les prestataires de soins de santé pour assurer la continuité des services maternels et néonatals en Guinée pendant la pandémie de covid-19. Nous allons interroger deux à trois membres du prestataire séniors des services sélectionnés à cet effet : par exemple comme le chef de service des sites, il y a également le surveillant général, le médecin consultant principal, ou encore la sage-femme maitresse.

Votre rôle dans l'étude

Votre décision de participer à cet entretien est totalement volontaire, vous avez le droit de sauter toute question à laquelle vous ne souhaitez pas répondre, et vous avez également le droit de mettre fin à cet entretien quand vous le souhaitez, ou de l'interrompre si d'autres tâches urgentes se présentent.

Risques et avantages possibles :

Cette étude ne présente pas de risque majeur pour les participants. Les informations que vous allez nous fournir seront confidentielles. Si vous vous sentez mal à l'aise au sujet de certaines questions, vous pouvez choisir de ne pas y répondre. Votre participation à cette étude va nous aider à documenter la préparation et riposte au COVID-19 dans les hôpitaux de références (maternité et de néonatologie) qui sont première ligne dans la riposte aux épidémies ou encore à la pandémie. Si vous acceptez de participer à l'étude, nous allons vous poser quelques questions.

Souhaitez-vous participer à cet entretien ?

- L'enquêteur enregistre la réponse du répondant : ___ OUI /___ NON
  Signature : _________________
- Consentez-vous à ce que j'enregistre cet entretien ? OUI ___ / ____NON
  Signature : _________________

Contact pour des questions ou problèmes

Si vous avez des questions par rapport à l'étude, vous pouvez contacter l'investigateur principal à l'adresse suivante : Alexandre DELAMOU, Tel: 628 594 765 ou e-mail: adelamou@maferinyah.org.

CERTIFICAT DE CONSENTEMENT

Noms et Prénom du participant ou de la participante :

……………………………………………………………………………………………………….....

J'ai lu les informations ci-dessus, ou elles m'ont été lues. J'ai eu l'occasion de poser des questions concernant ces renseignements et l'on m'a répondu à ma satisfaction. Je donne mon consentement volontairement pour participer à la présente étude, et je comprends que j'ai le droit de me retirer à tout moment sans aucune conséquence.

____ ____________________

Signature ou empreinte digitale du participant (e) Date

Si le ou participant(e) ne peut pas lire le formulaire lui-même, un témoin doit signer ici :

J'étais présent lorsque les avantages, les risques et les procédures ont été lus à la bénévole. Toutes les questions qu'elle a posées ont été répondues et ainsi elle a accepté de participer à l'étude.

_________________________ ____________________ ________________

Nom du témoin Signature Date

Je certifie que la nature et le but, les avantages potentiels et les risques éventuels associés à la participation à cette étude ont été expliqués à la personne ci-dessus et qu'elle a volontairement accepté de participer.

Guide de l'entretien semi-structuré

Ce guide d'entretien est destiné à être utilisé lors des entretiens réguliers avec les personnes interrogées dans les structures. Les questions exactes seront révisées régulièrement, et il y aura du temps pour noter d'autres développements, ou des questions que les répondants estiment importantes dans leur travail, même si elles ne sont pas couvertes par les questions de ce guide. Cet entretien ne devrait pas durer plus de 60 minutes. Le guide peut être légèrement différent d'un site d'étude à l'autre, en fonction des services fournis par chaque hôpital.

"Préparation et riposte au COVID-19 : une enquête sur les prestataires de soins de santé dans 3 sites (HNID, INSE et HRM) en Guinée"

- **Général :**

1) Depuis notre précèdent entretien (*mentionner la date*), y a-t-il des changements survenus dans votre structure de santé/service ? Si oui, de quoi d'agissait-il ? Quels sont les impacts de ces changements sur votre travail quotidien ?
    - Depuis le dernier entretien réalisé avec vous, y a-t-il eu de nouveaux changements dans le processus de consultation ou hospitalisation des usagers ?
        - Avez-vous modifié dans le service d'admission du service, l'organisation des lits, les zones d'attente dans la salle d'accouchement ou dans la salle ?
        - Frais facturés : y a-t-il eu un changement dans la tarification pour les usagers ?
        - Selon vous, les prestataires reçoivent-ils des primes ? Y a-t-il eu des changements dans ce domaine ?

2) Y a-t-il eu des changements (ex : comportement) survenus au niveau des usagers de votre service pendant cette période de COVID ? Si oui, parmi eux, y en avaient-ils qui étaient exigés par votre hôpital via-service ? Sont-ils tous respectés ?
    - Parmi les changements, y avait-il qui exigeait la séparation entre la mère et l'enfant ? Existe-il encore ? Etait-il difficile à appliquer ou à faire comprendre ?

3) Selon vous, qu'est-ce qui a changé pour le mieux depuis COVID-19 et que vous aimeriez conserver ?

4) Dans nos précédents entretiens, vous avez énuméré quelques problèmes que vous rencontré dans votre quotient liés à cette pandémie (*citer quelque uns*), ces problèmes existent-ils toujours ?
    - Si oui, comment continuez-vous à y faire face ? Si non, comment les avez-vous résolus ? De nouveaux problèmes ont-ils surgi ?

5) Que pensez-vous de la fréquentation du service par les usagers depuis le début de la pandémie ? Si baisse, quelle est votre perception ? Quel (les) est (sont) l'impact (s) sur le rendement du service ?

6) Y a-t-il eu un changement dans le type de patientes (femmes ou enfants) qui viennent dans votre structure pendant cette pandémie (depuis notre dernier entretien) ?

7) Si les usagers ne viennent pas dans votre service (qui est une structure de référence), selon vous, où partent-ils ?
    - Pendant cette période de COVID-19, certains prestataires ont-ils certaines formes de prestations de soins de santé telle-que les consultations aux téléphones, ou des

consultations à domicile ? Si oui, cette pratique continue-t-elle encore ? *Explorer en profondeur (du côté financier, logistique (connexion), avoir reçu des entrainements à propos de la prestation des soins par téléphone, etc.*

- Pensez-vous que financièrement la période de COVID profite aux prestations de santé privées (cliniques) ou consultations à domiciles ? Cette situation continue-t-elle encore ?

Des changements sont-ils survenus dans les domaines suivants depuis le début de cette pandémie au COVID-19 *(Suggestion de se concentrer sur 2-3 changements mentionnés avant)*

- Les processus et directives de soins, tels que le déclenchement du travail, l'option pour les césariennes, les options de soulagement de la douleur, la collecte de données de routine ou la déclaration après la crise COVID-19 ?
- Avez-vous fermé ou réduit certaines unités de votre service (unité de PF, CPN, accouchement, etc…), (unité de néonatologie, nutrition, etc…) ?
- De la consultation en tête-à-tête à la consultation virtuelle/téléphonique) :
- Parmi les unités de votre service, y a-t-il un changement : coté personnels de santé, rendement de chaque unité ?
  - Fournissez-vous des soins de routine comme vous le faisiez auparavant, certains éléments ont-ils été modifiés ou supprimés ? *(Interrogez séparément les soins prénatals, les accouchements et les soins postnatals, y compris l'allaitement, la durée du séjour, les soins aux nouveau-nés).*
  - Votre service autorise-t-il les accompagnants et/ou les visiteurs suite à la crise COVID-19 (accompagnants des femmes pour la maternité/ des enfants pour la néonat) ?
    - Si oui quels sont les règles (durant le travail, avant ou après l'accouchement / en période néonatale) ?
    - Qu'est ce qui a changé dans ces règles à cause du COVID-19 ?
  - Depuis le précèdent entretien, à la suite de la COVID-19, les politiques/règles suivantes ont-elles été modifiées ?
    - Maternité : où lavent-elles leur linge ? Où les personnes qui accompagnent les femmes dorment-elles, où séjournent-elles ?
    - Néonatalogie : où les personnes qui accompagnent les enfants dorment-elles, où séjournent-elles ?

8) Comment l'entretien (hygiène) du service est-il organisé ? Avez-vous fait des changements en cette période de pandémie

9) Les sources d'information que vous utilisez, sont-elles toujours fonctionnelles ? L'équipe est-elle capable de se réunir (staff par exemple) ? Quelle sont les difficultés qui y sont liées ?

10) Continuez-vous à recevoir les informations ou des directives du gouvernement guinéen ou du ministère de la santé concernant le COVID-19 ? Si oui, de quoi s'agissait-il ? En quoi cela vous a-t-il influencé ?

11) Le rôle de votre service (maternité/INSE) dans la riposte nationale/régionale au COVID-19 (rappeler la collaboration avec l'ANSS ou le CTPE), continuez-vous toujours ce soutient/rôle ? Si oui, quel type de soutient/rôle apportez-vous (décrire ce qui s'est passe en détails) ?

12) Avez-vous eu des cas suspects ou confirmés de COVID-19 chez les femmes enceintes au cours depuis notre dernier entretien ? Si oui : pouvez-vous me dire comment cela s'est passé ? Qu'avez-vous fait ?

13) Avez-vous eu des cas suspects ou confirmés de COVID-19 chez les nouveaux nés depuis notre dernier entretien ? Si oui : pouvez-vous me dire comment cela s'est passé ? Qu'avez-vous fait ?

14)

a) Quelle est l'évolution du niveau de stress du personnel de santé de votre service pendant cette période de COVID-19 ? Depuis notre dernier entretien à maintenant, quel est son niveau ? Comment les gens font-ils face à la situation ? Leurs principales préoccupations à l'heure actuelle ?

- B) Quelle est l'évolution du niveau de stress des usagers et parents qui fréquentent votre service pendant cette période de COVID-19 ? Depuis notre dernier entretien à maintenant, quel est son niveau ? Comment les gens font-ils face à la situation ? Leurs principales préoccupations à l'heure actuelle ?

15) Quels sont les équipements de protection individuelle (EPI) disponible dans votre service ? Quels sont les équipements que vous utilisez pour les soins prénatals, les accouchements par voie basse et par césarienne ?
- Est-ce que c'est possible aux prestataires de changer les EPI durant la journée ? avant de voir chaque patiente ?
- Les patientes doivent-elle porter les EPI pour les consultations ? Durant le travail (accouchement) ou au post partum ? Si oui, qui est responsable de chercher ces EPI aux patientes ?

16) Pouvez-vous me dire si votre service à une USIN ? *Si oui ? parler des changements survenus à l'USIN depuis notre dernier entretien ?*

17) Y a-t-il des changements dans la façon dont vous documentez le travail dans les dossiers des patients, les registres des patients, etc. depuis notre dernier entretien *(Ex : y a-t-il de nouveaux formulaires qui ont été introduits, ou utilisez-vous moins de formulaires, ou communiquez-vous des informations supplémentaires à des autorités telles que le ministère de la santé)* ?

18) Depuis notre dernier entretien, y a-t-il d'autres questions, préoccupations ou changements que vous aimeriez partager ?

Nous vous remercions du temps que vous nous avez accordé.
