# Supplementary material for: Perspectives and experiences of healthcare providers on the response to the COVID-19 pandemic in three maternal and neonatal referral hospitals in Guinea in 2020: a qualitative study
Source: BMC Health Serv Res. 2024 Feb 21;24:226. doi: 10.1186/s12913-024-10670-4 (PMC10882787; doi:10.1186/s12913-024-10670-4)
Supplement: Supplementary file 3 — Additional file 3. [file 12913_2024_10670_MOESM3_ESM.pdf]

Please find some quotes for each subthemes

Subthemes	***Quotes***
Reduced staffing levels	*"We received a circular from the Ministry of Health asking to release all the trainee staff, it was to avoid overloading the facility..., we also released early the trainees coming from schools of health and universities."* (Doctor N°1, Neonatology-INSE, Round 1)
	"in relation to the staff there has been a reduction, the staff has been reduced, all the trainees who were there have been released, just the regulars are left, we work in shifts, so far that's what continues." (Midwive N°2, HNID, Round 2)
Maintaining essential healthcare services	*"As far as the quality of the work in the delivery room or any other unit is concerned, the quality has not decreased because there are attendants who have an average of 15 to 20 years of experience. Although the number of staff has been reduced, everyone (still working) knows what to do. In fact, there were even women who have received care during COVID-19 who appreciated the quality of the work, especially in the delivery room."* (Doctor N°2, HRM, Round 1).
	"We're not like some departments that are sheltered from receiving emergencies directly, so we receive our emergencies, sometimes it's women who are evacuated in difficult conditions". (Doctor N°4, HNID, Round 2)
Suspension of staff daily meetings and introduction of a new information system for health providers	*"What we need to remember from this period [referring to the COVID-19 pandemic] for example this platform (referring to WhatsApp group) that is there, it is an innovation, ..."* (Doctor N°3, HNID, Round 4)
	"..., at the moment we don't do staff, we do the reporting through a WatsApp group. We've created a WatsApp group where we do the on-call report and there everyone reacts, criticisms and suggestions." (Doctor N°4, HNID, Round 2)
Co-management with COVID-19 treatment center (CTPE) for caesarean section cases among women who tested positive for SARS-CoV-2 virus	*"Being the only [functional] reference service at present, we are at the disposal of the ANSS [Agence National de Sécurité Sanitaire] and the infectious diseases services to support them whenever necessary [to perform the caesarean section on pregnant women who have tested positive for the COVID-19 virus] ... But these women do not stay with us here, yes, yes! After the surgery they and their babies return to the CTPE, ... CTPE of Donka."* (Doctor N°1, HNID, Round 1)
Management of infection prevention and control measures system on	*"We had to reinforce protection by applying barrier measures, especially in our departments ... Here we wash our hands, that's a habit already, even before COVID-19, before every act, after every act we did it, that's one, secondly we respect social distancing, thirdly we put on our masks, not only us and our*

the wards.	*patients who come to us, we oblige them to wear their masks."* (Midwife N°1, HRM, Round 1)
	"..., faced with the EBOLA epidemic, since that time, we had understood that protective equipment is really essential for any health worker. With COVID-19 ..., everyone is properly protected, and most often you'll find people wearing caps, bibs, gowns, in other words, your equipment, whether it's a doctor, a midwife, a nurse, even for the groundskeepers, we're really all outfitted at all times..." (Doctor N°2, HRM, Round 1)
COVID-19 Information effect on health care providers' daily work	*"Yes, of course we've received directives from the Ministry of Health, and we receive them regularly, including the various measures to be taken regarding the COVID19 response, directives for recognizing whether it's a confirmed case, suspected case, contact case, and so on."* (Doctor N°1, HRM, Round 1)
	"I don't receive any of this [Information from the National Response Committee] .... It's been a long time since I've received any statistics, otherwise there used to be statistics but they've slackened off. Otherwise, it was really good to keep people informed of developments". (Doctor N°3, HNID, Round 3)
Unavailability of PPE	*« the ministry never supplied us with PPE, the hospital sometimes supplies us (the maternity ward) with masks, not more… When you give 150 masks, it's not for half a day, we do not wear the masks for 2 or 3 days only… we wear them until they turn black". (*Doctor N°1, HNID, Round 4)
	"the problems we encounter here, it's the material side, it's the glove, the gloves are insufficient ...". (Nurse N°1, Neonatology-INSE, Round 1).
Lack of financial motivation	*"There were some people [referring to these provider colleagues] who refused to come to the ward, but we came, until now some don't come. We who agreed to come, we didn't get a pay raise, we didn't get a bonus [with a shrug]". (*Nurse N°2, Neonatology-INSE, Round 2).
Difficulties reducing crowding in the wards	*« This is one of the big problems of the ward. People have the culture of coming in large numbers with the patients, despite the COVID-19. We don't need three people to accompany one patient".* (Doctor N°3, HNID, Round 4).
	You know, in your department, especially neonatology, it's a very sensitive department, being the general supervisor, it's not easy to manage all these people who come in, especially from the mother's side, the fathers with the support staff, and especially in the African context, it's not easy. Tell me a little about how you manage to deal with all these people..." (Nurse N°1, Neonatology-INSE, Round 3)." (Nurse N°1, Neonatology-INSE, Round 3).
Fear of healthcare providers during COVID-19 response	*"A mother who has to spend 20,000 GNF [US$2.29] to bring her child here, if she uses 5,000 GNF [US$0.57] of phone credit to call the doctor and get advice from him, I think that's a gain for the mother. If it turns out that this child is not sick, we can treat him on an outpatient basis. Sometimes it's the transportation that tires them [the patient and the family], ...".*

	*(*Doctor N°1, Neonatology- INSE, Round 4).
	"since the time of EBOLA we are here, we come to the service, we have to take measures to avoid the infection… There were people who refused to come to the service, …, until now some don't come. We who accept to come, we are all afraid of being contaminated, but we take all the precautions not to be contaminated, it is not easy for us". (Nurse N°2, Neonatology- INSE, Round 2).
Perceived increase in severity of complications received	*"It is the users who no longer come, they have abandoned the referral hospital in favor of the [private] clinics. So, they use oxytocin, that the woman takes but she cannot give birth. She stays a long time and finally she comes to the [referral] hospital and this creates problems".* (Midwife N°2, HRM, Round 3).
	"..., private clinics where women give birth, they force deliveries, then they start first aid there. It doesn't work, they come back in really difficult conditions. In the neighborhoods, as you know there are a lot of clinics, it's uncontrolled, everyone does what they want, these problems are frequent." (Nurse N°3, Neonatology- INSE, Round 3).
COVID-19 cases among healthcare providers and parents of newborns	*« In all, I believe there have been 15 cases [referring COVID-19 cases among healthcare providers the HRM] ..., but in my maternity ward, we have had two cases that were managed at the CTPE [of Mamou]. So, this had an impact on the functioning of the service, as it created a certain psychosis."* (Doctor n°1, HRM, Round 3).
	"Since the start of this pandemic, we've had just one confirmed case; he was hospitalized and discharged cured". (Doctor N°1, Neonatology- INSE, Round 2).
